# Structural Integrity of Polymeric Components Produced by Additive Manufacturing (AM)—Polymer Applications

**DOI:** 10.3390/polym13244420

**Published:** 2021-12-16

**Authors:** Rui F. Martins, Ricardo Branco, Filippo Berto, Nuno Soares, Sebastião Bandeira

**Affiliations:** 1 UNIDEMI, Department of Mechanical and Industrial Engineering, Nova School of Science and Technology, Universidade NOVA de Lisboa, Campus de Caparica, 2829-516 Caparica, Portugal; 2 CEMMPRE, Department of Mechanical Engineering, Faculty of Sciences and Technology, University of Coimbra, Rua Luís Reis Santos, Pinhal de Marrocos, 3030-788 Coimbra, Portugal; ricardo.branco@dem.uc.pt; 3 Department of Mechanical and Industrial Engineering, Norwegian University of Science and Technology, 7491 Trondheim, Norway; filippo.berto@ntnu.no; 4 Department of Mechanical and Industrial Engineering, Nova School of Science and Technology, Universidade NOVA de Lisboa, Campus de Caparica, 2829-516 Caparica, Portugal; ng.soares@campus.fct.unl.pt (N.S.); s.bandeira@campus.fct.unl.pt (S.B.)

**Keywords:** polymers, additive manufacturing, structural integrity, PLA, Nylon-645

## Abstract

In the work presented herein, the structural integrity of polymeric functional components made of Nylon-645 and Polylactic acid (PLA) produced by additive manufacturing (Fused Deposition Modelling, FDM) is studied. The PLA component under study was selected from the production line of a brewing company, and it was redesigned and analyzed using the Finite Element Method, 3D printed, and installed under real service. The results obtained indicated that, even though the durability of the 3D printed part was lower than the original, savings of about EUR 7000 a year could be achieved for the component studied. Moreover, it was shown that widespread use of AM with other specific PLA components could result in even more significant savings. Additionally, a metallic hanger (2700 kg/m^3^) from the cockpit of an airplane ATR 70 series 500 was successfully redesigned and additively manufactured in Nylon 645, resulting in a mass reduction of approximately 60% while maintaining its fit-for-purpose. Therefore, the components produced by FDM were used as fully functional components rather than prototype models, which is frequently stated as a major constraint of the FDM process.

## 1. Introduction

Polymers, either in their natural or synthetic form, thermoplastic or thermosetting, can be considered cheap materials, characterised by low density and diverse characteristics of mechanical resistance, ductility, toughness, and viscoelasticity, to mention a few. Their use has been rising tremendously since the 1960s [1], replacing steel and glass, and through the introduction of an extensive list of new synthetic polymers in final products, generally obtained by injection molding. This growth was further enhanced in recent years with the massive use of the Fused Deposition Modelling (FDM) process.

FDM is one of the most popular additive manufacturing (AM) technologies for various engineering applications and was introduced commercially in the early 1990s by Stratasys Inc., USA. It is a Material Extrusion (ME) technique [2] that fabricates parts using a softened or melted thermoplastic filament form material continuously extruded through a nozzle, layer-upon-layer, based on 3D computer-aided design (CAD) instead of subtractive manufacturing methodologies [3]. In fact, since its invention, the impact of AM has continued to grow in both commercial and scholarly activities due to the processing of several types of polymers, and, more recently, metals. Therefore, this technology is shifting from prototyping to a dominant production industry, although limited so far to the non-large-scale production of components [4] and by the limited range of materials that can be processed [2]. In addition, the quality and reliability of solid-based FDM processed parts mainly depend on the careful selection of process variables, and is also limited by several drawbacks, including a lack of high mechanical strength and, in some cases, reduced dimensional accuracy (mainly due to high printing speeds) [2,5]. Thus, identifying the FDM process parameters that significantly affect the quality of FDM processed parts is essential, and researchers have explored several ways to improve mechanical properties and part quality using various experimental design techniques and concepts in recent years [6,7,8].

It is now evident that the mechanical properties of parts obtained by the FDM process are greatly influenced by various process parameters, such as infill density, infill patterns, extrusion and bed temperature, layer thickness/height, nozzle diameter, raster angle and width, air gap, shrinkage factor, part build orientation, machine calibration, and environmental factors such as humidity and temperature, just to mention a few [5,9,10,11]. It was also found in the literature that layer height is one of the most critical factors among those studied [5,12], and that extrusion temperature has also proved to be beneficial for enhancing the tensile strength of printed parts because of improved intralayer and interlayer adhesion (fiber-to-fiber bond strength), thereby reducing void density (porosity) [2,13]. Moreover, the ideal combination of parameters is challenging to achieve and is a complex process that influences the mechanical properties of the components and the final mesostructural characterization [12], which also depends on the material properties of the filament. Lower void densities, higher elastic moduli, and yield stresses were observed for lower layer heights [12]. The number of layers influences temperature gradient in the first layers, decreasing the void ratio and improving the strength of the bond, but can increase residual stresses due to the greater number of heating and cooling cycles [12]. A parametric study of the influence of FDM printing parameters revealed a decrease of more than 97% in void density when the layer height decreased from 0.3 to 0.1 mm, proving the paramount importance of this variable [12]. Additionally, FDM has proven to be a fast printing process that allows low part production cost and the use of a reasonable variety of materials, while presenting a poor surface finish and sometimes requiring support structures [14]. Surface roughness is generally reduced with a reduction in layer thickness during printing and/or by applying post-mechanical treatments like polishing/abrasive grinding, as well as by chemical methods such as acetone in vapor and liquid form [13]. Moreover, with the selection of an appropriate treatment time, optimum surface finish results are obtained without an unnecessary loss of dimensional accuracy [13] or a reduction in the strength of the printed parts. The parts produced in this investigation were not submitted to any surface improvement, either mechanical or chemical, since they were built with a medium layer thickness value without using support structures at critical locations.

**Figure 1:**
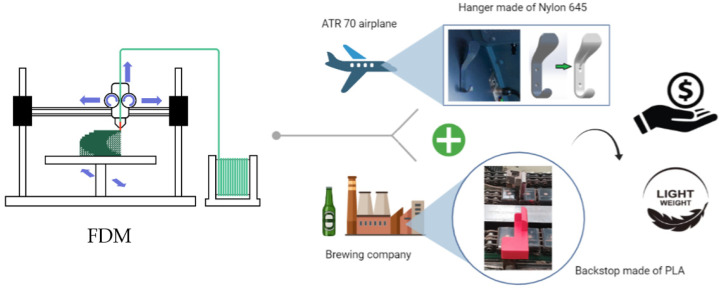
Graphical abstract on the use of functional polymeric lightweight components additively manufactured (FDM) in industrial applications.

Therefore, the work herein presented addressed some polymer applications, namely the structural integrity of polymeric components made of Nylon-645 and Polylactic acid (PLA) produced by additive manufacturing (FDM) (Figure 1). The PLA component under study was selected from the production line of a brewing company, and the Nylon component was chosen to replace a metallic hanger from the cockpit of an ATR 70 airplane. All parts were redesigned and analyzed using the Finite Element Method before being 3D printed and installed under real service. The results indicated that, even though the 3D-printed parts’ durability was lower than the original ones, savings could be achieved for the components under study. Moreover, it was shown that the widespread use of AM to other specific components of the brewing company could result in even more significant savings. Therefore, the components produced by FDM were used as fully functional components rather than prototype models, which is frequently stated as a major constraint of the FDM technique.

The manuscript starts with an introductory text about the use of polymers and AM, then presents two case studies of 3D-printed components that were used under real industrial applications. Finally, some conclusions were drawn.

## 2. Materials and Methods

This section describes the components studied and the methodologies employed during the investigation. Therefore, the materials that constitute the parts under study, their overall dimensions, and a description of the function of each component, to mention a few, are presented. Moreover, structural analyses of the components were carried out using the Finite Element Method. Therefore, boundary conditions, finite element mesh parameters, and loadings were addressed.

**Figure 2:**
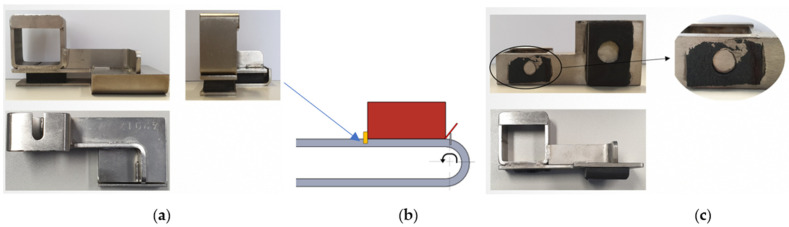
Component under study: (**a**) Overall view of the original backstop installed in the packaging line of a brewery company; (**b**) Description of the position and function of the backstop; (**c**) Typical failure of the backstop.

The first component under study is a backstop (Figure 2a) installed in a packaging line of a brewery company that works around 60,000 bottles of beer per hour, and that is used to fold paperboard around the bottles to produce the final delivery package (Figure 2b). The backstop is made of two metallic parts manufactured in an AISI 316 austenitic stainless steel connected by two glued pieces made of vulcanized rubber, and each of them costs EUR 250,00, it being necessary to replace approximately 33 backstops in the production line over the course of a year due to the occurrence of failures (Figure 2c). Moreover, during each stop, the maintenance time wasted to replace each backstop is approximately 15 min.

**Figure 3:**
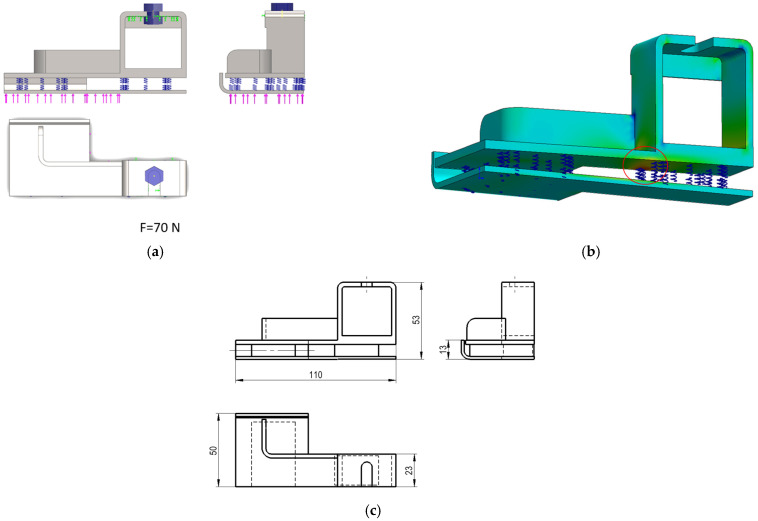
(**a**) Main views of the 3D-modelled backstop include: the force applied (70 N, arrows in pink), the pre-loaded bolt used, the fixed surface (in green) and the springs defined to simulate the vulcanized rubber pieces; (**b**) Detailed view of the critical region (vulcanized rubber) determined during the analysis of the original component; (**c**) Overall view of the main dimensions of the backstop (in millimeters).

During this investigation, the original backstop was three dimensionally modelled and structurally analyzed using a Finite Element (FE) commercially available software, namely SolidWorks Simulation. The vulcanized rubber pieces were substituted by springs with an appropriate elastic constant coefficient and tetrahedral solid finite elements were used to mesh the metallic parts. The number of distorted elements was equal to zero and the average element size was 3.18 mm, resulting in a total of 8699 elements and 17,108 nodes. In addition, the mechanical behavior of materials was considered linear, and a large displacement formulation was allowed during the finite element analysis (FEA), while a force of 70 N was applied to the backstop (Figure 3a). Moreover, fixtures were defined using a pre-loaded bolt that constrained a fixed surface (Figure 3a). The critical region of the component was localized at the vulcanized rubber position, coinciding with the fracture location that occurred in service (Figure 3b). The overall dimensions of the backstop can be found in Figure 3c.

**Figure 4:**
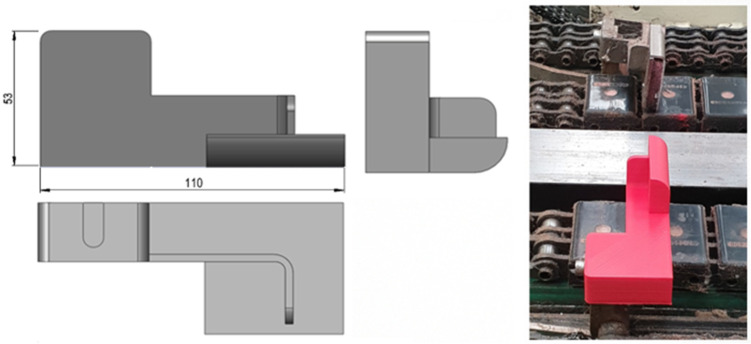
Redesigned backstop additively manufactured using PLA and installed in the packaging production line. Dimensions shown are in millimeters.

Considering the numerical results obtained in the finite element analysis carried out to the original component, a redesigned model of the backstop was conceived (Figure 4), FE analyzed, and additively manufactured using the FDM technique with a PLA filament diameter equal to 1.75 mm. An infill density of 100%, a shell thickness of 1.2 mm, a print speed of 60 mm/s, a printing temperature of 220 °C, a bed temperature equal to 45 °C, and a layer height of 0.2 mm were defined during the 3D printing of the new component, which was then installed in the production line (Figure 3). The production cost of each component was equal to EUR 3,23.

**Figure 5:**
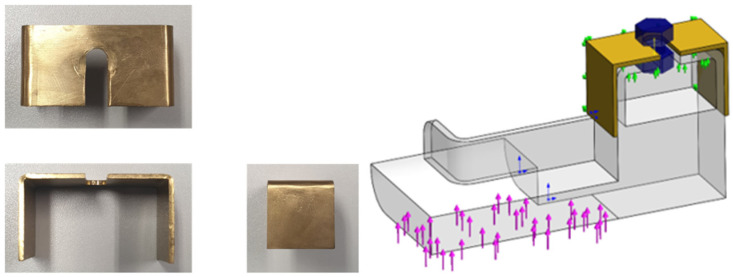
Overall view of the composite part; combination of the PLA component with a brass reinforcement with force applied (arrows in pink) 70 N, fixed fixtures (arrows in green) and surface under pre-load inserted by bolt (in blue); back of the folded thin sheet made of brass.

Moreover, in order to enhance the durability of the redesigned component at the vicinity of the pre-loaded bolt, a metallic strip sheet of brass was then folded and combined with the backstop made of PLA (Figure 5), allowing the composite part installed in the production line to endure 554 h, which corresponded to 1 ½ months in service. The critical region of this new assembly was determined through FEA and coincided with the fracture region verified in service (Section 3) [15].

**Figure 6:**
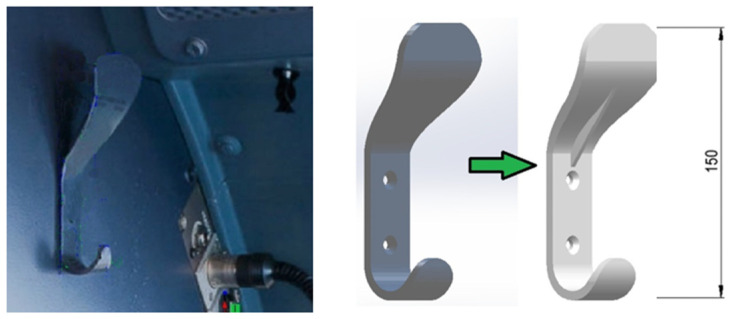
Metallic hanger from an airplane cockpit that was modelled and printed in Nylon 645. Dimensions shown are in millimeters.

**Figure 7:**
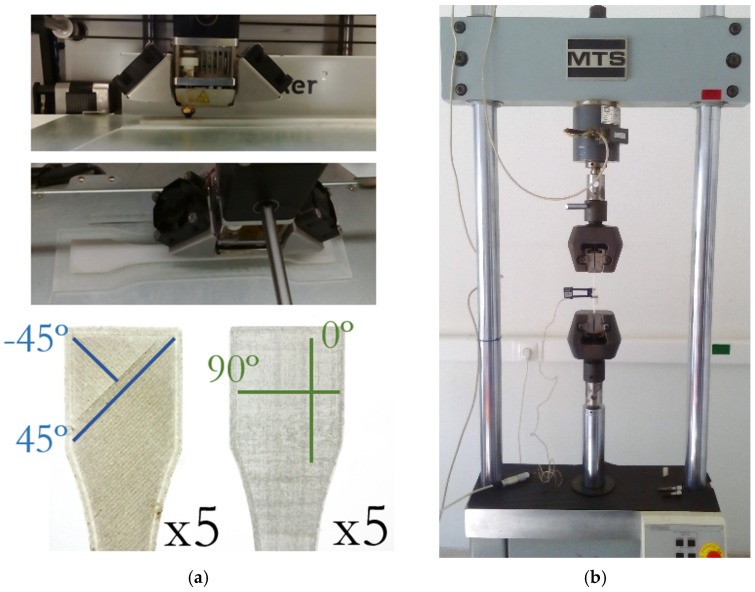
(**a**) Fiber-oriented specimens printed by AM; (**b**) MTS servo-hydraulic testing machine used to carry out the uniaxial tensile tests of the Nylon specimens printed by AM.

A second component considered was a metallic hanger (2700 kg/m^3^) from the cockpit of an airplane ATR 70, series 500 (Figure 6) [16]. A substitute hanger made of Nylon 645 (3 mm)—Taulman 3D [17] (1020 kg/m^3^) was additively manufactured aiming at reducing the mass of approximately 60% (Figure 6). The printing and the bed temperature were equal to 250 °C and 105 °C, respectively, while the printing velocity was about 40 mm/s. In addition, five ISO standard specimens [18,19], fiber oriented (−45°/45°), and another five specimens (0°/90°), were 3D printed (Figure 7a) to assess the quasi-static bulk mechanical properties of the Nylon 645 using an MTS servo-hydraulic testing machine (Figure 7b).

## 3. Results and Discussion

This section provides a description of the experimental results, their interpretation, as well as the experimental conclusions that can be drawn.

### 3.1. PLA Components

**Figure 8:**
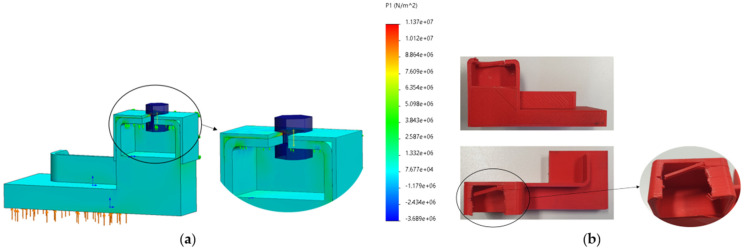
PLA redesigned backstop under study: (**a**) maximum principal stress, P1 [N/m^2^] induced in the composite component (11.37 MPa); (**b**) failure registered in the redesigned component after 554 h of operation, corresponding to 1 ½ months in real service.

The FE numerical simulations carried out in the mixed-material backstop shown in Figure 5 allowed for the calculation of a maximum principal stress value of 11.37 MPa in the PLA material under the prestressed bolt region (Figure 8a). The solid mesh used was a high-quality mesh, with an average element size of approximately 4.26 mm, and a total of 7682 elements and 13,355 nodes. The mechanical behavior of the brass material was modelled as linear elastic, with a Young’s modulus of 100 GPa, a Poisson’s ratio of 0.33, a yield stress of 240 MPa, and a tensile strength equal to 478 MPa; regarding the PLA, it was defined as possessing a linear elastic behavior with a Young’s modulus of 3000 MPa, a Poisson’s ratio of 0.35 and a tensile strength of 45 MPa. These properties defined for the polymeric material (PLA) compare well with a Young’s modulus of 3039 MPa, an ultimate strength of 48 MPa, and a strain at break of 2.5%, as described by Tan and his co-authors [2].

Moreover, the critical region numerically determined—where the maximum stress was calculated—was then experimentally confirmed in the component placed in the production line (Figure 8b) after 554 h of operation (corresponding to 1 ½ months in service), as the fracture surface due to fatigue loading coincided with the more stressed region.

The results indicate that, even though the 3D printed part’s durability is lower than the original one, savings of about EUR 6500 a year could be achieved for the component under study if it is produced by additive manufacturing (Table 1). These results already include the higher downtime associated with the replacement of the 160 expected redesigned backstops.

Additionally, widespread use of the additively manufacturing FDM process to produce other specific functional components of the brewing company, such as clamps, valve blockers, alarm pushbutton protectors, bottle selectors, and even sprockets, most of them not submitted to varying loadings (fatigue), could result in even more significant savings.

### 3.2. Nylon Component

**Figure 9:**
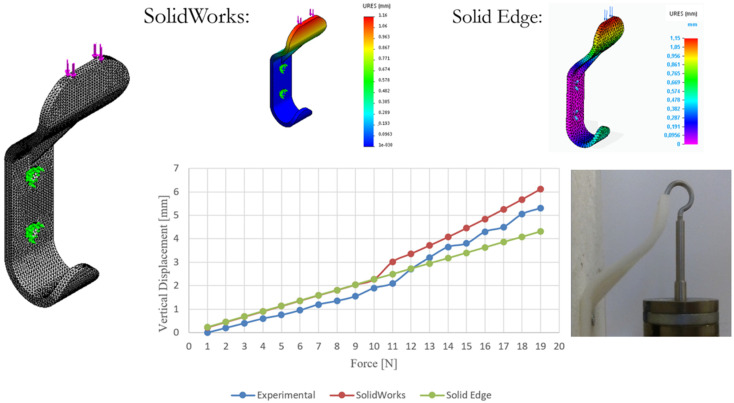
Comparison of the total displacement, URES (mm), obtained through experimental and numerical simulations in function of the deadweight downward force applied up to 19 N (calibrated masses).

**Figure 10:**
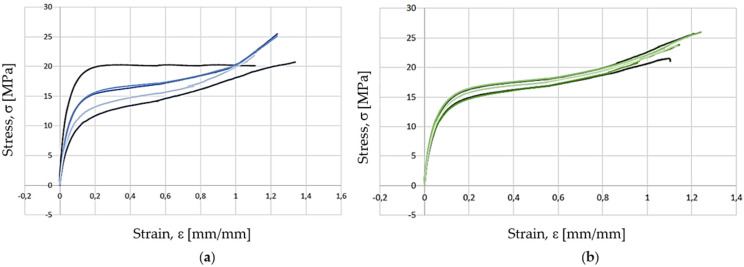
Mechanical properties of Nylon 645 under quasi-static loading: (**a**) Stress, s (MPa), versus strain, e (mm/mm) for −45°/45° fiber-oriented specimens; (**b**) stress, S (MPa), versus strain, e (mm/mm) for 0°/90° fiber-oriented specimens.

The hanger’s fit-for-purpose was verified by applying deadweight downward forces (calibrated masses) up to 19 N (Figure 9), which originated from a vertical displacement of the component measured through a calliper. The experimental displacements were compared with those calculated through computational simulations carried out in SolidWorks Simulation and SolidEdge (Figure 9). In addition, the material model specified during the numerical simulations was defined based on the bulk mechanical properties obtained in the quasi-static tests, namely an average Young’s modulus of about 450 MPa, a yield strength of 15 MPa, and a tensile strength equal to 25 MPa (Figure 10). In fact, concerning the bulk mechanical properties obtained for the printed specimens, these were very similar for the two configurations tested and almost independent from the filament orientation (Figure 10). Nevertheless, a slightly larger scatter was observed in the stress-strain curves obtained for the −45°/45° fiber-oriented specimens (Figure 10) regarding the determination of the yield stress and the tensile stress. Moreover, the experimental mechanical properties determined were in the range defined in [20], namely a tensile strength between 20–225 MPa, a yield stress range in 3.45–100 MPa, and a Young’s modulus of 210–16600 MPa.

Additionally, the final rupture (with fracture) was obtained only for two specimens, fiber-oriented produced along (0°/90°), that being the strain, e, very large, and up to 120% for almost all the tested specimens (Figure 10). The rupture naturally occurred for the two specimens produced along (0°/90°) once filaments oriented along 90° gave rise to weak intralayer bonding between filaments (and not by the filaments themselves), which were placed perpendicular to the primary load applied.

Finally, in Figure 9, a linear behavior is visible between the force applied and the vertical displacement measured up to 10 N, which changed experimentally for the range 11–19 N and in simulation from 10 N to 19 N; this resulted from the non-linear large displacement condition that was applied and detected experimentally with the SolidWorks Simulation software (Dassault Systèmes, Vélizy-Villacoublay, France) only. Nevertheless, this mechanical behavior does not compromise the function of the component. Therefore, the experimental and the numerical results were in good agreement and confirmed the possibility of using an AM lightweight, low price and structurally resistant hanger.

## 4. Conclusions

Several industrial applications of additively manufactured polymer components have been shown throughout the paper and the following conclusions can be drawn:

- Additive Manufacturing of polymers is a technology that shows great potential because it allows for the printing of very complex shapes, along with reduced waste of material and with reasonable mechanical properties;
- Nevertheless, Additive Manufacturing of polymers components as a functional part is still insignificant compared to injection molding components;
- However, with a careful selection of components and mechanical design, it is possible to guarantee the production of some parts by polymer additive manufacturing, allowing for less costly manufacturing parts and with fewer stocks;
- In the work herein presented, some examples were given for specific AM polymer components, allowing for significant money savings.
- Given the rapid pace of technological advancements, it is only a matter of time before AM becomes a feasible technology that complements traditional manufacturing techniques [2].

## Figures and Tables

**Table 1:** Cost analysis of the original part and the newly developed FDM part.

Component (Backstop)	Original	FDM
Production cost/component	-	EUR 3,23
Supply cost	EUR 250,00	-
Components needed/year	33	160
FDM cost	-	EUR 516,80
Supply cost	EUR 8250,00	-
Downtime (hours)	8,25	40
Maintenance cost	EUR 288,75	EUR 1400,00
Total Cost	EUR 8538,75	EUR 1916,80

## Data Availability

The data that support the findings of this study are available from the corresponding author upon reasonable request.

## References

[1] Ashby M.F. (2005). Materials Selection in Mechanical Design.

[2] Tan L.J., Zhu W., Zhou K. (2020). Recent Progress on Polymer Materials for Additive Manufacturing. Adv. Funct. Mater..

[3] Huang T.T., Wang S.G., He K.T. Quality control for fused deposition modeling based additive manufacturing: Current research and future trends. Proceedings of the First International Conference on Reliability Systems Engineering (ICRSE 2015).

[4] Nagarajan H.P.N., Haapala K.R. (2018). Characterizing the influence of resource-energy-exergy factors on the environmental performance of additive manufacturing systems. J. Manuf. Syst..

[5] Syrlybayev D., Zharylkassyn B., Seisekulova A., Akhmetov M., Perveen A., Talamona D. (2021). Optimisation of Strength Properties of FDM Printed Parts-A Critical Review. Polymers.

[6] Parandoush P., Lin D. (2017). A review on additive manufacturing of polymer-fiber composites. Compos. Struct..

[7] Fischer A., Rommel S., Bauernhansl T. New fiber matrix process with 3D fiber printer a strategic in-process integration of endless fibers using Fused Deposition Modeling (FDM). Proceedings of the IFIP TC 5 International Conference on Project Research On Leading-Edge Applications and Methods for Applied Information Technology, Digital Product and Process Development Systems.

[8] Laban O., Mahdi E., Samim S., Cabibihan J.J. (2021). A Comparative Study between Polymer and Metal Additive Manufacturing Approaches in Investigating Stiffened Hexagonal Cells. Materials.

[9] Mohamed O.A., Masood S.H., Bhowmik J.L. (2015). Optimization of fused deposition modeling process parameters: A review of current research and future prospects. Adv. Manuf..

[10] Solomon I.J., Sevvel P., Gunasekaran J. (2021). A review on the various processing parameters in FDM. Mater. Today Proc..

[11] Kumar S.R., Sridhar S., Venkatraman R., Venkatesan M. Polymer additive manufacturing of ASA structure: Influence of printing parameters on mechanical properties. Proceedings of the 2nd International Conference on Recent Trends in Metallurgy, Materials Science and Manufacturing (IMME).

[12] Garzon-Hernandez S., Garcia-Gonzalez D., Jérusalem A., Arias A. (2020). Design of FDM 3D printed polymers: An experimental-modelling methodology for the prediction of mechanical properties. Mater. Design.

[13] Gautam R., Idapalapati S., Feih S. (2018). Printing and characterisation of Kagome lattice structures by fused deposition modelling. Mater. Design.

[14] Kafle A., Luis E., Silwal R., Pan H.M., Shrestha P.L., Bastola A.K. (2021). 3D/4D Printing of Polymers: Fused DepositionModelling (FDM), Selective Laser Sintering (SLS), and Stereolithography (SLA). Polymers.

[15] Bandeira S. (2019). Applicability of the FDM Additive Manufacturing Process in the Production of Functional Parts Applicable in the Maintenance of the Filling Line of Sociedade Central de Cervejas. Master’s Thesis.

[16] Soares N. (2017). Verification of the Applicability of the Building Blocks Method to Parts Made by 3D Printing by Polymer Extrusion. Master’s Thesis.

[17] Nylon 645 Specifications, Taulman3d—High Strength Materials. https://taulman3d.com/nylon-645-spec.html.

[18] International Organization for Standardization (ISO) (2012). ISO 527-1:2012(E)—Plastics—Determination of Tensile Properties, Part 1: General Principles.

[19] International Organization for Standardization (ISO) (1997). ISO 527-4:1997—Plastics—Determination of Tensile Properties, Part 4: Test Conditions for Isotropic and Orthotropic Fibre-Reinforced Plastic Composites.

[20] MatWeb—Material Property Data, Overview of Materials for Nylon 6, Unreinforced. matweb.com.

